# Activation of NLRP3 inflammasomes in mouse hepatic stellate cells during *Schistosoma J.* infection

**DOI:** 10.18632/oncotarget.10044

**Published:** 2016-06-14

**Authors:** Nan Meng, Min Xia, Ya-Qi Lu, Mi Wang, Krishna M. Boini, Pin-Lan Li, Wang-Xian Tang

**Affiliations:** ^1^ Institute of Liver Diseases, Tongji Hospital, Tongji Medical College, Huazhong University of Science and Technology, Wuhan, China; ^2^ Department of Pharmacology and Toxicology, Virginia Commonwealth University, School of Medicine, Richmond, VA, USA

**Keywords:** NLRP3 inflammasome, hepatic stellate cell, cathepsin B, Schistosoma J., Pathology Section

## Abstract

The major pathological changes during *Schistosoma J.* infection are characterized by granulomatous inflammation in the liver, a cellular immune response to schistosomal egg antigens. The molecular mechanisms initiating or promoting this schistosomal granulomatous inflammation remain poorly understood. In the present study, we first demonstrated that in mice infected with *Schistosoma J*. for 6 weeks exhibited increased levels of IL-1β in liver, a major product of NLRP3 inflammasomes and collagen deposition around the eosinophilic granuloma with *Schistosoma J.* eggs, which was substantially attenuated by caspase-1 inhibitor, YVAD. This activation of the NLRP3 inflammasome occurred in hepatic stellate cells (HSCs), as shown by a marked increase in co-localization of IL-1β with HSCs marker, desmin. Using isolated, cultured mouse HSCs, we further explored the mechanisms by which soluble egg antigen (SEA) from *Schistosoma J.* activates NLRP3 inflammasomes. SEA induced the formation and activation of NLRP3 inflammasomes, which was associated with both redox regulation and lysosomal dysfunction, but not with potassium channel activation. These results suggest that NLRP3 inflammasome activation in HSCs may serve as an early mechanism to turn on the inflammatory response and thereby instigate liver fibrosis during *Schistosoma J.* infection.

## INTRODUCTION

Epidemiologic studies have indicated that schistosomiasis remains the second most common tropical disease with the high impact on human health due to blood flukes of the genus schistosoma. Over 249 million individuals in 73 countries around the world are infected with different types of schistosoma, and nearly 800 million people are under the threat of such infection [1–4]. Although various aetiological factors may lead to chronic liver injuries toward hepatic fibrosis or even cirrhosis such as drug abuses, alcoholism, hepatitis B and C viral infection, *Schistosoma J.* infection is one of the most common causes for hepatic fibrosis in some countries [5, 6]. It is well known that *Schistosoma J.* infection is characterized by pronounced immunological and inflammatory reactions against eggs deposited into liver or intestines, which eventually induce liver fibrosis, namely, schistosomiasis-associated liver fibrosis (SSLF) [7]. Some reports revealed that the treatment of efficacious schistosomicides cannot prevent the development of egg granuloma inflammation and hepatic fibrosis [7–9], which may be due to sustained pathological process like chronic inflammation. To date, the precise mechanisms that mediate this perpetual activation of inflammation around egg granulomas in the liver during *Schistosoma J.* infection remain poorly understood.

Recently, a new concept is emerging that the inflammasome, an intracellular inflammatory machinery, can switch on the inflammatory response of tissues to a variety of stimuli [10–12]. The nucleotide oligomerization domain (Nod)-like receptor family pyrin domain containing 3 (NLRP3) inflammasome is the most extensively investigated inflammasomes identified so far, and activation of the NLRP3 inflammasome may occur in response to a wide range of structurally dissimilar agonists, including pathogens, pore-forming toxins, environmental irritants, and endogenous damage-associated molecular patterns (DAMPs) [13, 14]. The NLRP3 inflammasome is formed into a large cytosolic multiprotein complex that typically consists of a sensor protein, NLRP3, the adaptor protein apoptosis-associated speck-like protein (ASC), and the pro-inflammatory caspase-1 [15, 16]. Upon stimulations, NLRP3 inflammasome components are assembled leading to the autocatalysis and activation of caspase-1, which is responsible for the maturation of pro-inflammatory cytokines, such as bioactive interleukin-1β (IL-1β) and interleukin-18 (IL-18) [10, 17–19]. Some studies reported that excessive production of IL-1β is involved in various systemic inflammatory diseases [20–22]. In the liver, the NLRP3 inflammasome was shown to mediate ischemia-reperfusion injury, and gene silencing of mouse *Nlrp3*gene protected the liver against ischemia-reperfusion injury through reduced IL-1βand IL-18 production and decreased inflammatory cell infiltration [23]. In addition, recent studies have indicated that besides inflammation instigation and inflammatory cell infiltration the activation of NLRP3 inflammasomes in fibroblasts including HSCs may result in the upregulation of transcription factors like transforming growth factor-β1 (TGF-β1), thereby causing myofibroblast-like differentiation and proliferation [24]. Moreover, there are reports that IL-1β contributes to localized inflammation in response to parasitic, bacterial or viral infections, which may be a critical mechanism for fibrogenesis with large increases in production of extracellular matrix (ECM) such as collagens [25–27]. These findings led us to wonder whether activation of the NLRP3 inflammasome in hepatic stellate cells (HSCs) triggers local inflammation in egg deposited area in liver and thereby switches on fibrogenic process leading to hepatic cirrhosis during *Schistosoma J.* infection.

The present study was designed to first determine whether *Schistosoma J.* infection *in vivo* or soluble egg antigen (SEA) stimulation *in vitro* can activate the NLRP3 inflammasome in HSCs. Then, we explored the molecular mechanisms mediating NLRP3 inflammasome activation upon SEA stimulation by three possible common pathways involved in inflammasomes activation, including lysosomal dysfunction and consequent cathepsin B release, the effects of reactive oxygen species (ROS) and potassium channel activation-associated K efflux. Our results demonstrated that NLRP3 inflammasomes serve as an intracellular inflammatory machinery in HSCs, which may turn on the inflammatory response and thereby instigate liver fibrosis during *Schistosoma J.* infection.

## RESULTS

### Mouse model of schistosomiasis-associated liver fibrosis (SSLF)

**Figure 1:**
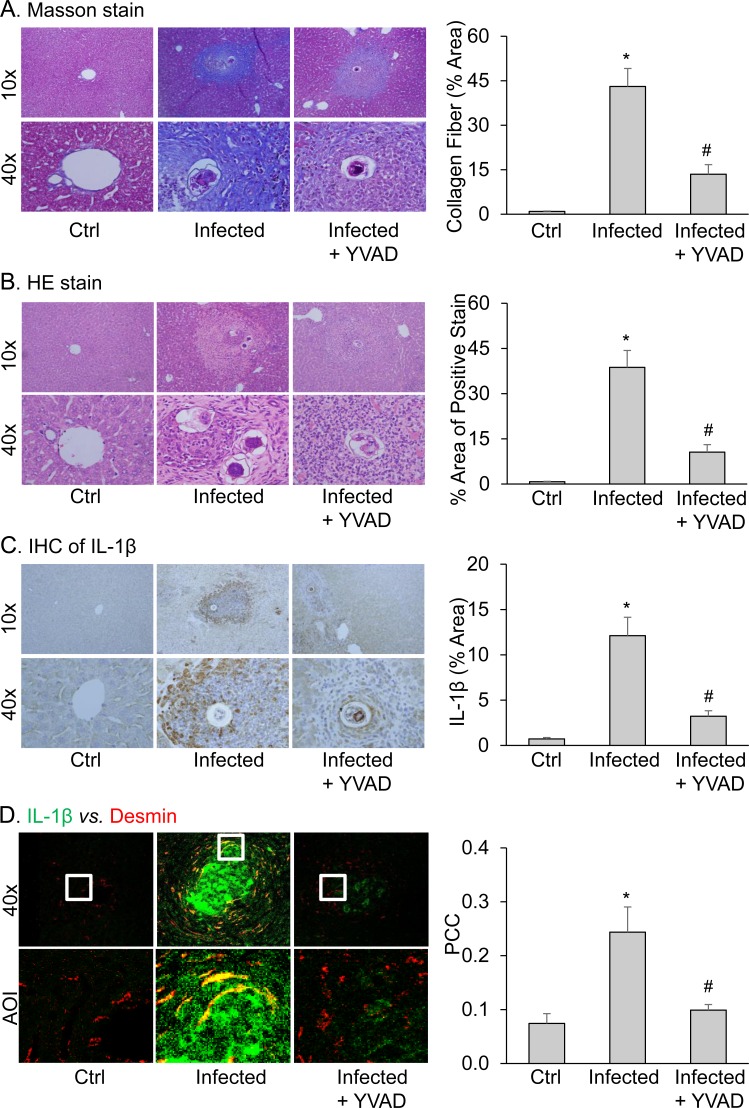
Pathological changes and IL-1β expression in livers from mice infected with *Schistosoma J.* for 6 weeks **A.** Representative images of Masson stain and quantitative analysis of positive stain of collagen fiber in portal area (*n* = 5). **B.** Representative images of HE stain (*n* = 4). **C.** Representative Immunohistochemical (IHC) images and quantitative analysis of positive stain of IL-1β in portal area (*n* = 5). **D.** Representative confocal fluorescence images show the co-localization of IL-1β with desmin and summarized data show co-localization coefficiency of IL-1β with desmin (*n* = 5). Ctrl, control; YVAD, caspase-1 inhibitor; Ctrl: Liver tissues from normal mice with minor fiber deposition around the vein; Infected: Infected mice showing the formation of liver fibrosis; YVAD + Infected: Infected mice treated with caspase-1 inhibitor YVAD (i.p. 1mg/kg/day). Data are expressed as means ± SEM. *p < 0.05 *versus* control group; #*p* < 0.05 *versus* infected group.

After 6 weeks of infection with *Schistosoma J.* cercariae, the mice had marked collagen deposition, fibrosis and inflammatory cells infiltration in the liver at the periphery of the eosinophilic granuloma, while *Schistosoma J.* eggs remained in the hepatic portal vein. These typical characteristics of SSLF were not observed in the livers of control mice (Figure 1A and 1B). These results confirmed a successful establishment of the SSLF model in *Schistosoma J.*-infected mice. By quantification of the area percentage positive for collagen staining in the liver, it was found that there were significantly more collagen depositions in the liver from *Schistosoma J.*-infected mice compared to that from control mice. Furthermore, these *Schistosoma J.*-induced increases in collagen deposition were markedly reduced when mice received a potent selective caspase-1 inhibitor, YVAD. Immunohistochemical studies demonstrated that no IL-1β staining was detected around the hepatic portal vein in the portal area from control mice (Figure 1C). However, the mice infected for 6 weeks with *Schistosoma J.* cercariae had a marked increase in IL-1β level around the *Schistosoma J.* eggs in the portal area. Such *Schistosoma J.* infection-induced IL-1β expression was also markedly reduced in mice pretreated with YVAD. Confocal microscopy showed increased co-localization of IL-1β with HSCs marker desmin around the *Schistosoma J.* eggs in the portal area (Figure 1D), which was attenuated in mice receiving YVAD.

### SEA-induced the NLRP3 inflammasome formation and increased caspase-1 activity and IL-1β production in hepatic stellate cells (HSCs)

**Figure 2:**
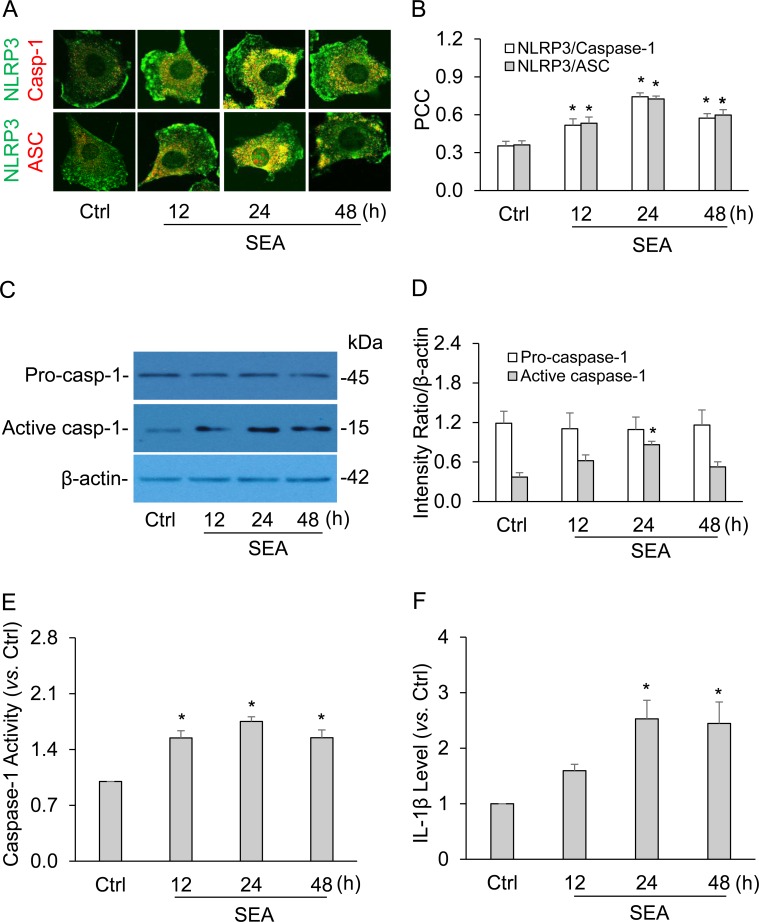
SEA induced the formation and activation of NLRP3 inflammasome in HSCs at different time points **A.** Representative confocal fluorescence images show the co-localization of NLRP3 with ASC or caspase-1. **B.** Summarized data show co-localization coefficiency of NLRP3 with ASC or caspase-1 (*n* = 5). **C.** and **D.** Representative Western blot documents and summarized data show the effects of SEA (50 μg/ml for 24h) on the expression of pro-caspase-1, cleaved caspase-1 and β-actin in HSCs (*n* = 6). **E.** and **F.** Data summary shows relative caspase-1 activity (n = 6) and IL-1β production compared with control (*n* = 6). SEA, soluble egg antigen from *Schistosoma J.*; HSCs, hepatic stellate cells; Pro-casp1, pro-caspase-1; Cle-casp1, cleaved caspase-1. Data are expressed as means ± SEM. *p < 0.05 *versus* untreated control group.

With the use of cultured mouse HSCs, we first characterized the formation of NLRP3 inflammasomes by confocal microscopy. As shown in Figure 2A, the co-localization of NLRP3 with ASC or caspase-1 increased upon SEA stimulation at different time points (12, 24 and 48 hours), indicating the aggregation or assembly of these inflammasome molecules. The Pearson correlation coefficient of NLRP3 with ASC or caspase-1 was summarized to represent the co-localization efficiency (Figure 2B). Such co-localization of NLRP3 molecules suggests the formation of NLRP3 inflammasomes in HSCs when they were exposed to SEA stimulation. The maximum co-localization level was observed after 24-hour treatment of SEA in HSCs, therefore, this duration and dose of SEA treatment were used in the rest of experiments, if not otherwise mentioned. We also found that SEA significantly increased the level of active or cleaved caspase-1, indicating the activation of NLRP3 inflammasome because increased cleavage of pro-caspase-1 into bioactive caspase-1 is one of the important functions of activated NLRP3 inflammasomes (Figure 2C and 2D). However, SEA did not have effects on pro-caspase-1 level despite its consumption during inflammasome activation, which may be related to its large content or up-regulation for protein synthesis. Consistently, biochemical analysis showed that SEA significantly increased caspase-1 activity (Figure 2E) and IL-1β release (Figure 2F) in HSCs.

### Effects of mouse *Nlrp3* gene silencing on SEA-induced NLRP3 inflammasome activation

**Figure 3:**
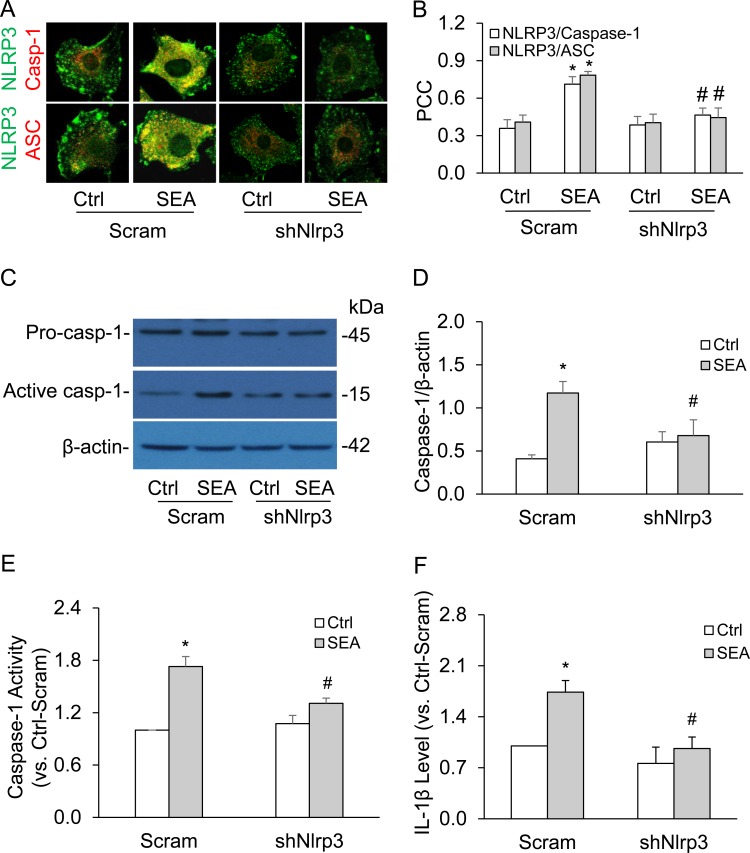
*Nlrp3* gene silencing inhibited SEA-induced NLRP3 inflammasome activation in HSCs **A.** Representative confocal fluorescence images show the co-localization of NLRP3 with ASC or caspase-1. **B.** Summarized data show co-localization coefficiency of NLRP3 with ASC or caspase-1 (*n* = 5). **C.** and **D.** Representative Western blot documents and summarized data (*n* = 5). **E.** and **F.** Data summary shows relative caspase-1 activity (*n* = 5) and IL-1β production compared with control (*n* = 5). Scram, scrambled shRNA; *shNlrp3*, *Nlrp3* shRNA. Data are expressed as means ± SEM. *p < 0.05 *versus* scrambled control group; #*p* < 0.05 *versus* scramble + SEA group.

Knockdown of mouse *Nlrp3* mRNA level by *Nlrp3* shRNA in HSCs markedly inhibited SEA-induced co-localization of NLRP3 with ASC or caspase-1 (Figure 3A and 3B) and attenuated SEA-increased level of cleaved caspase-1 (Figure 3C and 3D). Consistent with these findings, *Nlrp3* gene silencing almost completely blocked SEA-induced caspase-1 activity (Figure 3E) and IL-1β production in HSCs (Figure 3F).

### Effects of caspase-1 inhibition on SEA-induced NLRP3 inflammasome activation

**Figure 4:**
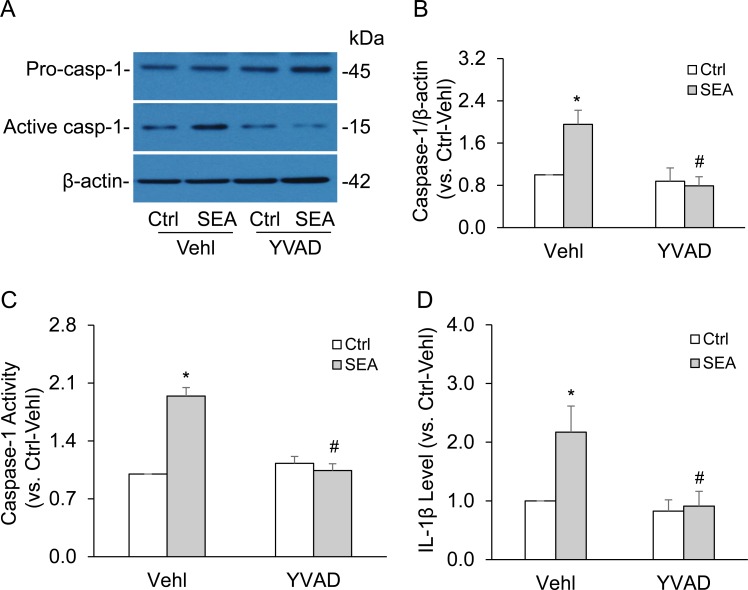
Caspase-1 inhibitor YVAD abolished SEA-induced caspase-1 activation and IL-1β production in HSCs HSCs were stimulated without or with SEA in the presence of PBS (Vehl: vehicle), caspase-1 inhibitor Ac-YVAD-CMK (YVAD, 10 μg/ml, Cayman Chemical). **A.** and **B.** Representative Western blot documents and summarized data (*n* = 6). **C.** and **D.** Data summary shows relative caspase-1 activity (*n* = 7) and IL-1β production compared with control (*n* = 5). Vehl: vehicle; YVAD, caspase-1 inhibitor. Data are expressed as means ± SEM. *p < 0.05 *versus* untreated control group; #*p* < 0.05 *versus* SEA group.

We also tested the effect of caspase-1 inhibition on SEA-induced caspase-1 activation and IL-1β production *via* NLRP3 inflammasomes. It was found that blockade of caspase-1 activity by YVAD almost completely abolished SEA-induced increases in cleaved caspase-1 level as shown by Western blot analysis (Figure 4A and 4B) and substantially suppressed SEA-enhanced caspase-1 activity and IL-1β production in HSCs (Figure 4C and 4D). This caspase-1 inhibition did not have effect on the formation of NLRP3 inflammasomes because of its effect only on active caspase-1, downstream of inflammasome assembling.

### Mechanisms by which NLRP3 inflammasomes are activated by soluble egg antigen (SEA)

**Figure 5:**
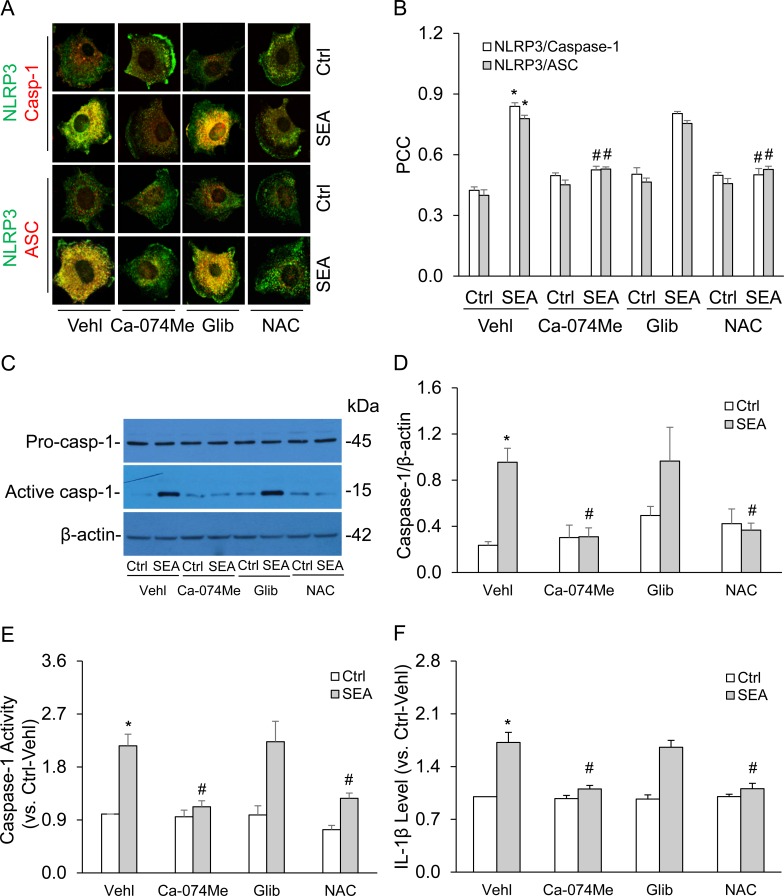
Effects of cathepsin B inhibition, potassium channel blockade or ROS scavenging on SEA-induced formation and activation of NLRP3 inflammasomes in HSCs HSCs were stimulated without or with SEA in the presence of PBS (Vehl: vehicle), cathepsin B inhibitor Ca-074Me (5 μM, Sigma), potassium channel blocker glibenclamide (Glib, 10 μm, Sigma) or ROS scavenger N-acetyl-L-cysteine (NAC, 10 μM, Sigma). **A.** Representative confocal fluorescence images show the colocalization of NLRP3 with ASC orcaspase-1. **B.** Summarized data show colocalization coefficiency of NLRP3 with ASC or caspase-1 (*n* = 5). **C.** and **D.** Representative Western blot documents and summarized data (*n* = 6). **E.** and **F.** Data summary shows relative caspase-1 activity (*n* = 6) and IL-1β production (*n* = 5). Vehl: vehicle; Ca-074Me, cathepsin B inhibitor; Glib, potassium channel blocker glibenclamide; NAC, ROS scavenger N-acetyl-L-cysteine. Data are expressed as means ± SEM. *p < 0.05 *versus* untreated control group; #*p* < 0.05 *versus* SEA group.

We further examined how SEA stimulates activation of NLRP3 inflammasomes in HSCs using inhibitors of 3 common pathways activating this inflammasome. It was found that inhibition of both cathepsin B activity and ROS release in HSCs markedly attenuated SEA-induced co-localization of NLRP3 with ASC or caspase-1 (Figure 5A and 5B). Consistently, inhibition of cathepsin B activity and scavenging of ROS release by using cathepsin B inhibitor, Ca-074Me and N-acetyl-L-cysteine(NAC) almost completely blocked SEA-induced increases in cleaved caspase-1 level, caspase-1 activity and IL-1β production in HSCs (Figure 5C-5F). In contrast, Glibenclamide (Glib), a K channel blocker had no significant effect on SEA-induced NLRP3 inflammasome formation and activation. In additional experiments, we tested whether inhibition of NADPH oxidase attenuates SEA-induced caspase-1 activation and IL-1β production in HSCs. It was found that inhibition of NADPH oxidase by gp91ds-tat significantly attenuated the SEA-induced increases in cleaved caspase-1 levels and IL-1β production in HSCs, implicating a role for NADPH oxidase in inflammasome activation (Supplemental Figure 1).

### Effects of cathepsin B gene silencing on SEA-induced NLRP3 inflammasome activation in HSCs

**Figure 6:**
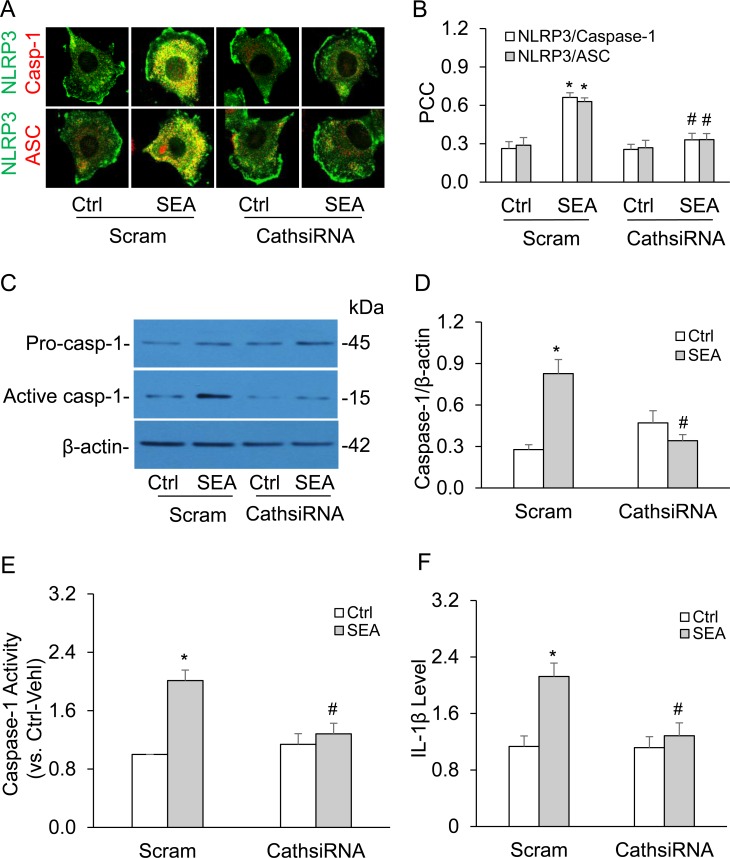
Cathepsin B gene silencing inhibited SEA-induced NLRP3 inflammasome activation in HSCs **A.** Representative confocal fluorescence images show the co-localization of NLRP3 with ASC or caspase-1. **B.** Summarized data show co-localization coefficiency of NLRP3 with ASC or caspase-1 (*n* = 5). **C.** and **D.** Representative Western blot documents and summarized data (*n* = 6). **E.** and **F.** Data summary shows relative caspase-1 activity (*n* = 6) and IL-1β production compared with control (*n* = 5). Scram, scrambled siRNA; CathsiRNA, Cathepsin B siRNA. Data are expressed as means ± SEM. *p < 0.05 *versus* scrambled control group; #*p* < 0.05 *versus* scramble + SEA group.

Since our previous studies have shown that SEA may increase NADPH oxidase activity and enhanced production of ROS from activated NADPH oxidase activate inflammasomes [4, 28], the present study focused on how SEA acts through altered lysosome permeability and cathepsin B activity. We demonstrated that silencing of cathepsin B gene markedly inhibited SEA-induced co-localization of NLRP3 with ASC or caspase-1 (Figure 6A and 6B) and reduced SEA-enhanced cleavage of pro-caspase-1 (Figure 6C and 6D), caspase-1 activity (Figure 6E) and production of IL-1β (Figure 6F). These results further confirmed that cathepsin B is indeed involved in SEA-induced NLRP3 inflammasome formation and activation in HSCs.

### SEA increased lysosome membrane permeability leading to enhanced cathepsin B release and activity in HSCs

**Figure 7:**
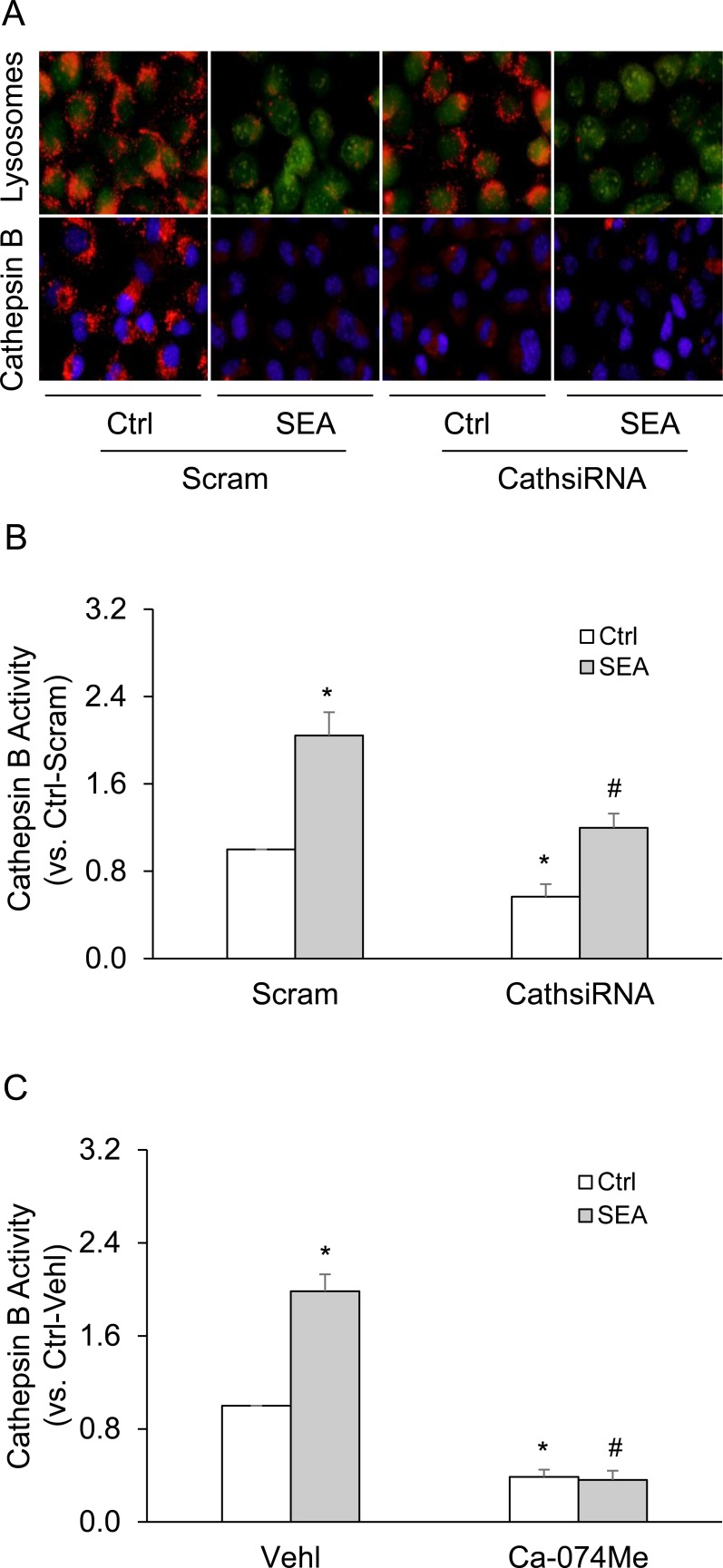
SEA stimulation increased lysosome membrane permeability, subsequent cathepsin B release and activity in HSCs **A.** Representative fluorescence images show the fluorescence intensity of lysosome and cathepsin B (*n* = 5). **B.** and **C.** Data summary shows relative cathepsin B activity compared with control (*n* = 5). Scram, scrambled siRNA; CathsiRNA, Cathepsin B siRNA; Ca-074Me, cathepsin B inhibitor (5 μM, Sigma). Data are expressed as means ± SEM. *p < 0.05 *versus* scrambled control group or untreated control group; #*p* < 0.05 *versus* scramble + SEA group or SEA group.

In these experiments, living HSCs were stained with lysomotropic agent, acridine orange, which accumulates in acidic organelles such as lysosome with red fluorescence, but shows green fluorescence in a neutral environment such as nucleus or cytoplasm. This fluorescence changes can be used to directly observe changes in the lysosome membrane permeability. Lysosomal alkalization induced by lysosome membrane permeability leads to decreased red fluorescence from acridine orange. In control HSCs, lysosomes were visualized as red puncta with high fluorescence intensity. However, SEA stimulation resulted in decreased fluorescence intensity of red puncta (Figure 7A). Similarly, these HSCs were stained with a fluorescent cathepsin B substrate z-Arg-Arg-cresyl violet for cathepsin B activity, and with hoechst staining for the cell nuclei. Silencing cathepsin B genes decreased fluorescence intensity of cathepsin B substrate in lysosomes, and SEA stimulation lead to decrease in fluorescence intensity of cathepsin B substrate in these lysosomes (Figure 7A), which is an indicative for the leakage of cathepsin B into cytoplasm. Silencing cathepsin B genes or cathepsin B inhibitor Ca-074Me also reduce cathepsin B activity, while SEA stimulation enhanced cathepsin B activity in control HSCs, but in HSCs treated with cathepsin B siRNAs or cathepsin B inhibitor Ca-074Me (Figure 7B and 7C).

### Effects of lysosome membrane stabilizer on SEA-induced NLRP3 inflammasome activation

**Figure 8:**
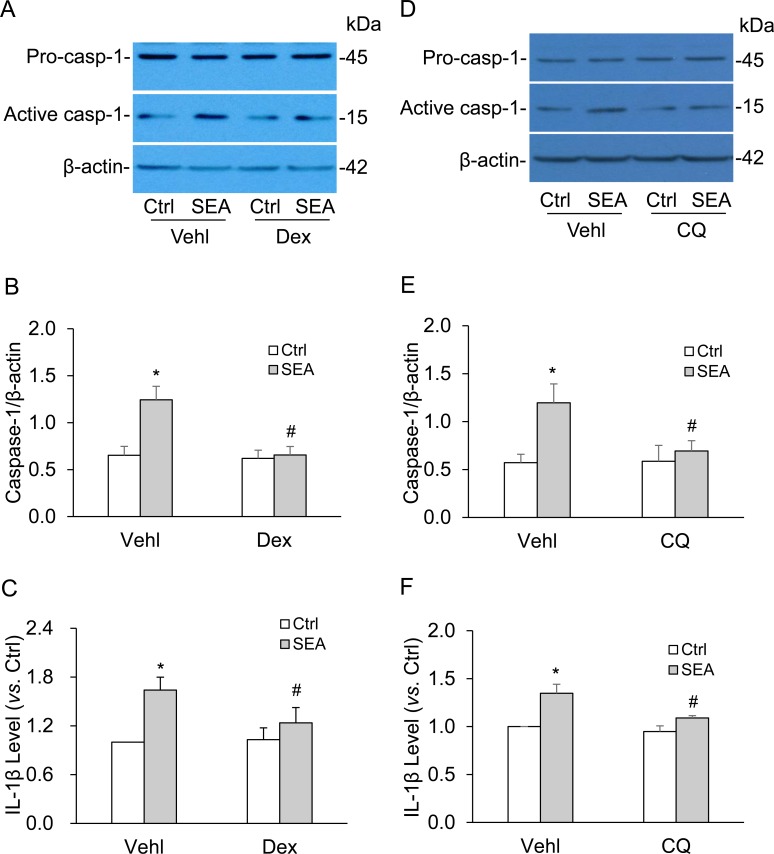
Lysosome membrane stabilizer abolished the SEA-induced caspase-1 activation and IL-1β production in HSCs HSCs were stimulated with or without SEA in the presence of PBS (Vehl: vehicle), lysosome membrane stabilizer dexamethasone (Dex, 100 μm, Sigma) or chloroquine (CQ, 20 μm, Sigma). **A.** and **B.**, **D.** and **E.** Representative Western blot documents and summarized data (*n* = 5). **C.** and **F.** Data summary shows IL-1β production (*n* = 4). Vehl: vehicle; DEX: Dexamethasone, CQ: Chloroquine. Data are expressed as means ± SEM. *p < 0.05 versus untreated control group; #*p* < 0.05 *versus* SEA group.

We also tested the effect of lysosome membrane stabilizer on SEA-induced caspase-1 activation and IL-1β production *via* NLRP3 inflammasomes. It was found that lysosome membrane stabilizer dexamethasone (Dex) or chloroquine (CQ) significantly abolished the SEA-induced increases in cleaved caspase-1 levels and IL-1β production in HSCs (Figure 8).

## DISCUSSION

In the present study, we demonstrated that *Schistosoma J.* infection or soluble egg antigens (SEA) from *Schistosoma J.* eggs induced the formation and activation of NLRP3 inflammasomes in hepatic stellate cells (HSCs) in both *in vitro* and *in viv*o. In the mice infected for 6 weeks with *Schistosoma J.* cercariae, confocal microscopy studies showed increased co-localization of IL-1β with HSCs marker desmin around the *Schistosoma J.* eggs in the portal area, which can be inhibited by caspase-1 inhibitor YVAD. This NLRP3 inflammasome activation in HSCs upon SEA is associated with both redox regulation and lysosomal dysfunction-related increases in cathepsin B release and activity.

To determine whether NLRP3 inflammasome activation occurs in HSCs from the liver of mice infected with *Schistosoma J*. and whether this inflammasome activation is implicated in the development of schistosomiasis-associated liver fibrosis (SSLF), we first produced a SSLF mouse model with *Schistosoma J*. cercariae. It was found that the IL-1β level and collagen deposition were markedly increased at the periphery of the eosinophilic granuloma with *Schistosoma J.* eggs in the liver of mice infected with cercariae. In mice receiving YVAD, a caspase-1 inhibitor, the fibrogenic pathology and the increases in IL-1β level were substantially attenuated. These results suggest that during *Schistosoma J.* infection inflammasome activation is accompanied with fibrogenic changes in the liver, which provides the first evidence that the development of SSLF may be associated with caspase-1 activation *via* inflammasome. In previous studies, IL-1β was found to contribute to local inflammation in response to parasitic, bacterial or viral infections [25–27], and the inflammasome was reported to be involved in the complex signaling pathway driving fibrosis under different pathological conditions, and the role of inflammasomes has been related to their inflammatory or non-inflammatory effects [24]. In this regard, there are some reports that the formation and activation of NLRP3 inflammasomes in fibroblasts including HSCs may produce IL-1β or IL-18, resulting in the upregulation of transforming growth factor-β1 (TGF-β1) and collagens and also causing myofibroblast differentiation into active fibroblast to trigger or promote fibrogenesis [24]. In mice lacking NLRP3 or ASC, the development of liver fibrosis induced by CCl4 and TAA was significantly impaired and MSU-induced upregulation of collagen I and TGF-βcould not be observed in ASC^−/−^ HSCs [29]. These results support the view that NLRP3 inflammasome activation in HSCs contributes to the onset or development of local inflammation and fibrosis in the liver during *Schistosoma J.* infection.

To determine whether the formation and activation of NLRP3 inflammasomes in HSCs are due to stimulation of *Schistosoma J*. eggs as main pathogenic factor of local inflammation in the liver and to explore related molecular mechanisms mediating NLRP3 inflammasome activation, we performed a series of experiments in primary cultures of HSCs. It was found that exposure of HSCs to SEA even at 24 hours led to remarkable formation and activation of NLRP3 inflammasomes, as shown by aggregation of NLRP3 with ASC orcaspase-1 and increases in caspase-1 activity and IL-1β release from HSCs. When HSCs were pretreated with caspase-1 inhibitor, YVAD or with mouse *Nlrp3* shRNA, the activation of NLRP3 inflammasomes was almost completely blocked. Based on these results, it is clear that the NLRP3 inflammasomes are present and functioning in HSCs and that SEA is a potent stimulus for their activation. Increased production of IL-1β and other cytokines or chemokines during this inflammasome activation may induce local inflammation and ultimately lead to SSLF. There is substantial evidence that during *Schistosoma J.*infection it is their eggs that stimulate local inflammation in the liver and that such local liver inflammation is importantly implicated in the development of consequent hepatic cirrhosis [30, 31]. However, the molecular mechanisms that turn on the local liver inflammation and fibrogenesis remains poorly understood. The results from the present study indicate that the NLRP3 inflammasome as an intracellular inflammatory machinery in HSCs can be activated by *Schistosoma J.* eggs or its extracts to trigger the local inflammatory response and ultimately induce liver fibrogenesis.

This present study further explored the mechanisms by which SEA stimulate HSCs to activate NLRP3 inflammasomes. Since there are three major mechanisms or pathways reported to activate NLRP3 inflammasomes upon different pathogen associated molecular patterns and damage associated molecular patterns, namely, increased generation of reactive oxygen species, enhanced lysosome membrane permeability and elevated K^+^ efflux [28, 32–35], we first tested which of these pathways are involved in SEA-induced NLRP3 inflammasome activation. Using blockers or inhibitors of individual pathway, we found that SEA-induced NLRP3 inflammasome formation and activation in HSCs were significantly attenuated or abolished by ROS scavenger, N-acetyl-L-cysteine (NAC) and cathepsin B inhibitor, Ca-074Me, but not by potassium channel blocker, glibenclamide (Glib). These results suggest that SEA as *Schistosoma J.* egg extracts is able to activate NLRP3 inflammasomes in HSCs at least *via* two main reported pathways involving increased ROS and frustrated lysosomes and enhanced cathepsin B activity [28, 32]. In regard to the role of ROS in activation of NLRP3 inflammasomes, there are increasing evidence that ROS act on its target molecules TXNIP leading to a dissociation of thioredoxin and thereby increasing NLRP3 binding to ASC. This results in a rapid assembling of NLRP3 inflammasome activating caspase-1 to produce IL-1β and other pathogenic factors to trigger inflammatory response [36–41] or directly promote fibrogenesis in some types of cells such as HSCs. In a recent study, we have demonstrated that *Schistosoma J.* infection or SEA activated NADPH oxidase (NOX) to produce O_2_^.−^ and inhibition NOX activity or silencing NOX subunit gene blocked SEA-induced O_2_^.−^ production and prevented liver fibrosis and related fibrogenic changes in HSCs [4]. Taken together, these results establish a new working model to induce SSLF, where *Schistosoma J.* infection or SEA activate NOX in HSCs to produce ROS and thereby activate NLRP3 inflammasomes, leading to caspase-1 activation and consequent production of different factors that instigate the local inflammatory response or induce fibrogenic changes in the liver. This NOX-derived O_2_^.−^ may serve as a kindling ROS to activate inflammasomes, which may further lead to the bonfire production of ROS due to inflammatory response including immune cells infiltration and respiratory burst. It is clear that the activation of inflammasomes is a critical step for chronic injurious action of *Schistosoma J.* infection.

We performed more experiments to further confirm the role of increased lysosome membrane permeability and cathepsin B activation. It was demonstrated that SEA stimulation increased lysosomal permeabilization and thereby led to release of cathepsin B from these frustrated lysosomes in HSCs. This released cathepsin B was activated in the cytoplasma, which is thought to activate NLRP3 inflammasomes through an undescribed mechanism. These results represent the first experimental evidence that SEA may make lysosomes of HSCs frustrated and thereby release and activate NLRP3 inflammasomes. In other cell types, however, some pathological stimulates such as lysosome dysfunction resulting in NLRP3 inflammasome activation [42–46]. Our recent studies have demonstrated that a bacterial wall extract, LCWE also stimulate NLRP3 inflammasome by increases in lysosome membrane permeability and cathepsin B activation in endothelial cells, which may be a fundamental mechanism triggering coronary arteritis [32]. It is believed that the frustrated lysosome and associated cathepsin B release and activation are one of the important mechanisms mediating NLRP3 inflammasome activation during bacterial and parasite infections.

In summary, the present study showed that the formation and activation of NLRP3 inflammasomes occurred in HSCs during *Schistosoma J.* infection or upon stimulation of SEA from *Schistosoma J.* eggs. The activation of NLRP3 inflammasomes is mechanistically implicated in the development of local inflammation and fibrogenesis in the liver. SEA was found to increase ROS, which may be a mechanism mediating this inflammasome activation, and it may also make lysosome of HSCs frustrated to release and activate cathepsin B, resulting in the NLRP3 inflammasome activation. Our results suggest that the activation of NLRP3 inflammasomes in HSCs may be a critical early mechanism developing local inflammation and fibrosis in liver during *Schistosoma J.* infection. In perspective, targeting the inflammasome activation and associated molecular mechanisms may be a novel therapeutic strategy for prevent and treatment of hepatic cirrhosis during and after *Schistosoma J.* infection.

## MATERIALS AND METHODS

### Animals and reagents

BALB/C mice (6 weeks of age, male) were obtained from a Schistosomiasis Control Station in Hubei province, China. *Schistosoma J.* egg antigen [SEA, 0.01 g/ml in phosphate-buffered saline (PBS)] is a gift from the Hubei Schistosomiasis Control Station. SEA was diluted to the working concentration in Dulbecco's modified Eagle's medium (DMEM) supplemented with 2% fetal bovine serum (FBS) before use.

### Animal model development

The schistosomiasis-associated liver fibrosis (SSLF) model was established by abdominal infection with *Schistosoma J.* cercariae according to the protocols described in previous studies [47, 48]. In brief, mice in the model group were percutaneously infected with *Schistosoma J*. cercariae by placing a glass slide carrying 20 ±2 of them (in non-chlorinated water) on the abdomen of each mouse for 15 min. In another group, mice were injected with YVAD (i.p. 1 mg/kg/day), a caspase-1 inhibitor prior to *Schistosoma J*. infection for 1 week and after such infection for 6 weeks. Control group mice were treated with non-chlorinated water containing no cercariae. All mice were maintained for 6 weeks under pathogen-free conditions with free access to food and water. At the end point, these mice were sacrificed, and liver tissues were harvested for morphological examinations, confocal and other analyses. All animal experiments were performed according to the Guide for the Care and Use of Laboratory Animals of the Chinese Council on Animal Care.

### Hepatic stellate cells (HSCs) isolation and culture

Mouse HSCs were prepared by the discontinuous density gradient centrifugation technique as previously described [47] and some minor modifications were made to increase success rate as we described previously [4, 31]. The collected cells were cultured in DMEM (Gibco, Carlsbad, CA) containing 10% FBS (Gibco) in humidified 95% air and 5% CO2 mixture at 37°C. The cell viability, as measured by a Trypan Blue exclusion assay, was approximately 90%.HSCs were treated with SEA (50 μg/ml) for indicated hours. HSCs were characterized and confirmed as previously described [4, 31]. Because the optimum response of inflammasome activation was observed after 24-hour SEA stimulation, the same treatment was used in all experiments of the current study, if not otherwise mentioned. To inhibit caspase-1 activity in HSCs, the prior treatment with Ac-YVAD-CMK (YVAD, 10 μg/ml, Cayman Chemical) for 30 min was performed. Similarly, before SEA stimulation in HSCs, the prior treatment with cathepsin B inhibitor Ca-074Me (5 μM, Sigma), potassium channel blocker glibenclamide (Glib, 10 μM, Sigma) or ROS scavenger N-acetyl-L-cysteine (NAC, 10 μM, Sigma) for 30 min was conducted.

### Masson staining

Masson staining is a commonly used methods to observe collagen fiber deposition, which was performed according to the previous study [49]. After staining, 5 different fields under microscope with 200x magnifications were randomly selected from each tissue slides for analysis, and the data were presented by the area percentage of positive staining with blue color, from quantitation using Image Pro Plus 6.0 software (Media Cybernetics Inc, United States). In addition, HE staining was performed to analyze the liver structure according to the protocol described previously [50].

### Immunohistochemistry

Liver tissues were fixed in 4% (v/v) paraformaldehyde (PFA) in PBS and embedded with paraffin, which were then sliced into tissue sections (4 μM) and mounted on glass slides. These tissue slides were stained with goat anti-IL-1β antibody (1:100, R&D Systems) overnight at 4°C after a 20 min wash with 3% H_2_O_2_ and 30 min blocking with 10% serum and then probed with anti-goat Ig-G second antibody labeled with HRP according to the protocols described previously [32, 40]. Negative controls were prepared without the primary antibodies. The area percentage of the positive staining were calculated in Image Pro Plus 6.0 software.

### Immunofluorescence microscopy

To confirmNLRP3 inflammasome formation in HSCs in mouse liver tissue, frozen tissue slides were stained with goat anti-IL-1β antibody (1:100, R&D Systems) and rabbit anti-desmin antibody (1:100, Thermo Fisher) overnight at 4°C after a 30 min blocking with 10% serum and then probed with Alexa 488- or Alexa-555-labeled secondary antibodies (1:500, Invitrogen). Similarly, cells were incubated with goat anti-NLRP3 antibody (1:200, Abcam), rabbit anti-ASC antibody (1:200, Santa Cruz), or rabbit anti-caspase-1 antibody (1:200, Santa Cruz) as we described previously [40, 51]. Co-localization of IL-1β *vs*. desmin, NLRP3 *vs*. ASC or caspase-1 was calculated in Image Pro Plus 6.0 software, and the co-localization coefficient was calculated as the Pearson's correlation coefficient.

### Western blot analysis

Proteins from cell lysates were denatured with SDS buffer and boiled for 5 minutes. Samples were run on a SDS-PAGE gel, transferred onto polyvinylidene difluoride (PVDF) membrane, and blocked with 5% milk. Membranes were probed with the following primary antibodies overnight at 4°C: rabbit anti-caspase-1 (1:400, Santa Cruz) and rabbit anti-β-actin (1:5000, Santa Cruz). Then, they were incubated with goat anti-rabbit-HRP IgG (1:5000, Santa Cruz) for 1 hour at room temperature. Bands were detected by chemiluminescence techniques after washing three times and visualized on Kodak Omat X-ray film. The intensity of the specific bands was calculated using ImageJ software version 1.44p.

### Assay of Caspase-1 activity and quantitation of IL-1β level

Control and SEA-treated HSCs were washed twice with PBS, scraped in lysis buffer to extract proteins and measure caspase-1 activity according to manufacturer's instructions of a commercially available caspase-1 activity assay kit (Biovision). Cell supernatant was harvested and IL-1β level was measured by using a mouse IL-1β ELISA kit (R&D Systems) according to the manufacturer's instructions.

### Nucleofection

Transfection of shRNA plasmids was performed according to the protocol of nucleofection as we described previously [32]. After nucleofection, HSCs were cultured in DMEM that contained 10% FBS for 24 hour, and then treated with SEA (50 μg/ml) or vehicle without SEA for corresponding experiments.

### RNA interference of cathepsin B gene

Cathepsin B small interference RNAs (siRNAs) were commercially available (Santa Cruz), and the scrambled RNA has been confirmed as nonsilencing double-stranded RNA and was used as a control in this present study. SiRNA transfection was performed with the siLentFect Lipid Reagent (Bio-Rad, USA) according to the protocol described by the manufacturer.

### Analysis of lysosome membrane permeability and cathepsin B leakage detection

Hepatic stellate cells (HSCs) were cultured in eight-well chamber slides and treated without or with soluble egg antigen (SEA). After SEA treatment for 24h, cells were measured lysosome membrane permeability and cathepsin B in HSCs by using a commercially available CV-cathepsin B detection kit (Enzo life technology) according to the protocol described by the manufacturer. HSCs in chamber slides were immediately analyzed and photographed using a fluorescence microscope (Fluoview FV1000, Olympus, Japan).

### Cathepsin B activity assay

After SEA treatment for 24 h, cells were washed twice with PBS, scraped in lysis buffer to extract proteins, and measured cathepsin B activity in HSCs by using a commercially available cathepsin B activity fluorometric assay kit (Biovision) according to the protocol described by the manufacturer.

### Statistical analysis

Data are presented as means ± SEM. The student's test was used to evaluate the differences between two groups. One-way or two-way ANOVA was used based on the data to detect significant differences among multiple groups of data. *P* < 0.05 was considered statistically significant. All statistical analyses were performed using SigmaStat 11.0.

## SUPPLEMENTARY MATERIAL



## References

[1] Hotez PJ, Kamath A (2009). Neglected tropical diseases in sub-saharan Africa: review of their prevalence, distribution, and disease burden. PLoS Negl Trop Dis.

[2] Elmorshedy H, Bergquist R, El-Ela NE, Eassa SM, Elsakka EE, Barakat R (2015). Can human schistosomiasis mansoni control be sustained in high-risk transmission foci in Egypt?. Parasit Vectors.

[3] Steinmann P, Keiser J, Bos R, Tanner M, Utzinger J (2006). Schistosomiasis and water resources development: systematic review, meta-analysis, and estimates of people at risk. Lancet Infect Dis.

[4] Wang M, Abais JM, Meng N, Zhang Y, Ritter JK, Li PL, Tang WX (2014). Upregulation of cannabinoid receptor-1 and fibrotic activation of mouse hepatic stellate cells during Schistosoma J. infection: role of NADPH oxidase. Free Radic Biol Med.

[5] McManus DP, Gray DJ, Li Y, Feng Z, Williams GM, Stewart D, Rey-Ladino J, Ross AG (2010). Schistosomiasis in the People's Republic of China: the era of the Three Gorges Dam. Clin Microbiol Rev.

[6] Wallace K, Burt AD, Wright MC (2008). Liver fibrosis. Biochem J.

[7] Gryseels B, Polman K, Clerinx J, Kestens L (2006). Human schistosomiasis. Lancet.

[8] Cioli D, Pica-Mattoccia L (2003). Praziquantel. Parasitol Res.

[9] Southgate VR, Rollinson D, Tchuem Tchuente LA, Hagan P (2005). Towards control of schistosomiasis in sub-Saharan Africa. J Helminthol.

[10] Strowig T, Henao-Mejia J, Elinav E, Flavell R (2012). Inflammasomes in health and disease. Nature.

[11] Wen H, Ting JP, O'Neill LA (2012). A role for the NLRP3 inflammasome in metabolic diseases—did Warburg miss inflammation?. Nat Immunol.

[12] Boini KM, Xia M, Abais JM, Li G, Pitzer AL, Gehr TW, Zhang Y, Li PL (2014). Activation of inflammasomes in podocyte injury of mice on the high fat diet: Effects of ASC gene deletion and silencing. Biochim Biophys Acta.

[13] Ratsimandresy RA, Dorfleutner A, Stehlik C (2013). An Update on PYRIN Domain-Containing Pattern Recognition Receptors: From Immunity to Pathology. Front Immunol.

[14] Chen Y, Pitzer AL, Li X, Li PL, Wang L, Zhang Y (2015). Instigation of endothelial Nlrp3 inflammasome by adipokine visfatin promotes inter-endothelial junction disruption: role of HMGB1. J Cell Mol Med.

[15] de Zoete MR, Palm NW, Zhu S, Flavell RA (2014). Inflammasomes. Cold Spring Harb Perspect Biol.

[16] Schroder K, Tschopp J (2010). The inflammasomes. Cell.

[17] de Zoete MR, Flavell RA (2013). Interactions between Nod-Like Receptors and Intestinal Bacteria. Front Immunol.

[18] Martinon F, Burns K, Tschopp J (2002). The inflammasome: a molecular platform triggering activation of inflammatory caspases and processing of proIL-beta. Mol Cell.

[19] Hornung V, Latz E (2010). Intracellular DNA recognition. Nat Rev Immunol.

[20] Martinon F, Tschopp J (2004). Inflammatory caspases: linking an intracellular innate immune system to autoinflammatory diseases. Cell.

[21] Church LD, Cook GP, McDermott MF (2008). Primer: inflammasomes and interleukin 1beta in inflammatory disorders. Nat Clin Pract Rheumatol.

[22] Venugopal R, Galam L, Cox R, Fukumoto J, Cho Y, Parthasarathy PT, Lockey RF, Kolliputi N (2015). Inflammasome Inhibition Suppresses Alveolar Cell Permeability Through Retention of Neuregulin-1 (NRG-1). Cell Physiol Biochem.

[23] Zhu P, Duan L, Chen J, Xiong A, Xu Q, Zhang H, Zheng F, Tan Z, Gong F, Fang M (2011). Gene silencing of NALP3 protects against liver ischemia-reperfusion injury in mice. Hum Gene Ther.

[24] Artlett CM, Thacker JD (2015). Molecular activation of the NLRP3 Inflammasome in fibrosis: common threads linking divergent fibrogenic diseases. Antioxid Redox Signal.

[25] Kolb M, Margetts PJ, Anthony DC, Pitossi F, Gauldie J (2001). Transient expression of IL-1beta induces acute lung injury and chronic repair leading to pulmonary fibrosis. J Clin Invest.

[26] Osuka A, Hanschen M, Stoecklein V, Lederer JA (2012). A protective role for inflammasome activation following injury. Shock.

[27] Thomay AA, Daley JM, Sabo E, Worth PJ, Shelton LJ, Harty MW, Reichner JS, Albina JE (2009). Disruption of interleukin-1 signaling improves the quality of wound healing. Am J Pathol.

[28] Abais JM, Xia M, Zhang Y, Boini KM, Li PL (2015). Redox regulation of NLRP3 inflammasomes: ROS as trigger or effector?. Antioxid Redox Signal.

[29] Watanabe A, Sohail MA, Gomes DA, Hashmi A, Nagata J, Sutterwala FS, Mahmood S, Jhandier MN, Shi Y, Flavell RA, Mehal WZ (2009). Inflammasome-mediated regulation of hepatic stellate cells. Am J Physiol Gastrointest Liver Physiol.

[30] Wilson MS, Mentink-Kane MM, Pesce JT, Ramalingam TR, Thompson R, Wynn TA (2007). Immunopathology of schistosomiasis. Immunol Cell Biol.

[31] Liu P, Wang M, Lu XD, Zhang SJ, Tang WX (2013). Schistosoma japonicum egg antigen up-regulates fibrogenesis and inhibits proliferation in primary hepatic stellate cells in a concentration-dependent manner. World J Gastroenterol.

[32] Chen Y, Li X, Boini KM, Pitzer AL, Gulbins E, Zhang Y, Li PL (2015). Endothelial Nlrp3 inflammasome activation associated with lysosomal destabilization during coronary arteritis. Biochim Biophys Acta.

[33] Zhou R, Yazdi AS, Menu P, Tschopp J (2011). A role for mitochondria in NLRP3 inflammasome activation. Nature.

[34] Mariathasan S, Weiss DS, Newton K, McBride J, O'Rourke K, Roose-Girma M, Lee WP, Weinrauch Y, Monack DM, Dixit VM (2006). Cryopyrin activates the inflammasome in response to toxins and ATP. Nature.

[35] Lamkanfi M (2011). Emerging inflammasome effector mechanisms. Nat Rev Immunol.

[36] Cruz CM, Rinna A, Forman HJ, Ventura AL, Persechini PM, Ojcius DM (2007). ATP activates a reactive oxygen species-dependent oxidative stress response and secretion of proinflammatory cytokines in macrophages. J Biol Chem.

[37] Dostert C, Petrilli V, Van Bruggen R, Steele C, Mossman BT, Tschopp J (2008). Innate immune activation through Nalp3 inflammasome sensing of asbestos and silica. Science.

[38] Tschopp J, Schroder K (2010). NLRP3 inflammasome activation: The convergence of multiple signalling pathways on ROS production?. Nat Rev Immunol.

[39] Zhou R, Tardivel A, Thorens B, Choi I, Tschopp J (2010). Thioredoxin-interacting protein links oxidative stress to inflammasome activation. Nat Immunol.

[40] Xia M, Boini KM, Abais JM, Xu M, Zhang Y, Li PL (2014). Endothelial NLRP3 inflammasome activation and enhanced neointima formation in mice by adipokine visfatin. Am J Pathol.

[41] Fukumoto J, Fukumoto I, Parthasarathy PT, Cox R, Huynh B, Ramanathan GK, Venugopal RB, Allen-Gipson DS, Lockey RF, Kolliputi N (2013). NLRP3 deletion protects from hyperoxia-induced acute lung injury. Am J Physiol Cell Physiol.

[42] Boya P, Kroemer G (2008). Lysosomal membrane permeabilization in cell death. Oncogene.

[43] Guicciardi ME, Leist M, Gores GJ (2004). Lysosomes in cell death. Oncogene.

[44] Gasse P, Riteau N, Charron S, Girre S, Fick L, Petrilli V, Tschopp J, Lagente V, Quesniaux VF, Ryffel B, Couillin I (2009). Uric acid is a danger signal activating NALP3 inflammasome in lung injury inflammation and fibrosis. Am J Respir Crit Care Med.

[45] Duewell P, Kono H, Rayner KJ, Sirois CM, Vladimer G, Bauernfeind FG, Abela GS, Franchi L, Nunez G, Schnurr M, Espevik T, Lien E, Fitzgerald KA (2010). NLRP3 inflammasomes are required for atherogenesis and activated by cholesterol crystals. Nature.

[46] Goncalves VM, Matteucci KC, Buzzo CL, Miollo BH, Ferrante D, Torrecilhas AC, Rodrigues MM, Alvarez JM, Bortoluci KR (2013). NLRP3 controls Trypanosoma cruzi infection through a caspase-1-dependent IL-1R-independent NO production. PLoS Negl Trop Dis.

[47] Schafer S, Zerbe O, Gressner AM (1987). The synthesis of proteoglycans in fat-storing cells of rat liver. Hepatology.

[48] Chen BL, Zhang GY, Yuan WJ, Wang SP, Shen YM, Yan L, Gu H, Li J (2011). Osteopontin expression is associated with hepatopathologic changes in Schistosoma japonicum infected mice. World J Gastroenterol.

[49] Huang DK, Zhang YX, Man SQ, Yu FZ, Shen JJ (2012). Comparison of collagen fiber staining between Van-Gieson staining and Masson trichrome staining of hepatic specimens in mice with Schistosoma japonicum infection [Article in Chinese]. Zhongguo Xue Xi Chong Bing Fang Zhi Za Zhi.

[50] Zhang RZ, Qiu H, Wang N, Long FL, Mao DW (2015). Effect of Rheum palmatum L. on NF-kappaB signaling pathway of mice with acute liver failure. Asian Pac J Trop Med.

[51] Boini KM, Xia M, Koka S, Gehr TW, Li PL (2016). Instigation of NLRP3 inflammasome activation and glomerular injury in mice on the high fat diet: role of acid sphingomyelinase gene. Oncotarget.

