# Supplementary material for: Activation of NLRP3 inflammasomes in mouse hepatic stellate cells during Schistosoma J. infection
Source: Oncotarget. 2016 Jun 14;7(26):39316–31. doi: 10.18632/oncotarget.10044 (PMC5129935; doi:10.18632/oncotarget.10044)
Supplement: Supplementary file 1 [file oncotarget-07-39316-s001.pdf]

Activation of NLRP3 inflammasomes in mouse hepatic stellate cells during *Schistosoma J.* infection

Supplementary Material

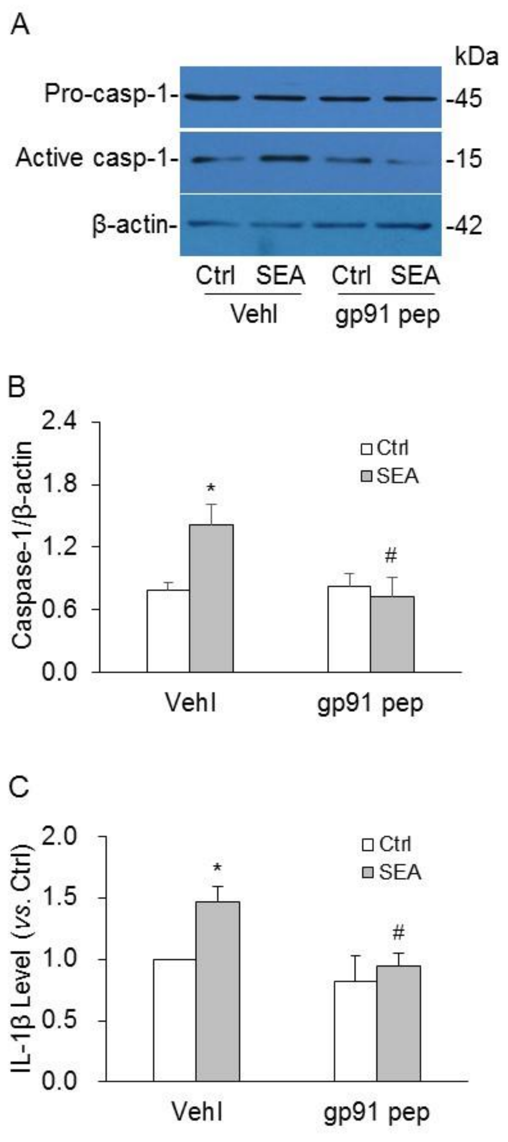

Supplemental Figure 1. NADPH oxidase inhibitor gp91 pep abolished SEA-induced caspase-1 activation and IL-1β production in HSCs. HSCs were stimulated without or with SEA in the presence of PBS (Vehl: vehicle), NADPH oxidase inhibitor gp91 pep (20 μm). **A and B.** Representative Western blot documents and summarized data (n=5). **C.** Data summary shows IL-1β production compared with control (n=4). Vehl: vehicle; gp91 pep, NADPH oxidase

inhibitor. Data are expressed as means ± SEM. *$p < 0.05$ versus untreated control group; #$p < 0.05$ versus SEA group.
